# Utilizing an Augmented Reality Headset to Accurately Quantify Lower Extremity Function in Parkinson’s Disease

**DOI:** 10.3390/s26041216

**Published:** 2026-02-13

**Authors:** Andrew Bazyk, Colin Waltz, Ryan D. Kaya, Eric Zimmerman, Joshua D. Johnston, Benjamin L. Walter, Anson B. Rosenfeldt, Mandy Miller Koop, Jay L. Alberts

**Affiliations:** 1 Center for Neurological Restoration, Neurological Institute, Cleveland Clinic, 9500 Euclid Ave., Cleveland, OH 44195, USA; bazyka2@ccf.org (A.B.); kayar@ccf.org (R.D.K.); zimmere3@ccf.org (E.Z.); walterb7@ccf.org (B.L.W.); 2 Department of Biomedical Engineering, Lerner Research Institute, Cleveland Clinic, 9500 Euclid Ave., Cleveland, OH 44195, USA; johnstj@ccf.org (J.D.J.); rosenfa2@ccf.org (A.B.R.); koopm@ccf.org (M.M.K.)

**Keywords:** Parkinson’s disease, markerless motion capture, gait biomechanics, augmented reality, stepping in place

## Abstract

Subjective, imprecise evaluation of lower extremity function hinders the effective treatment of gait impairments in Parkinson’s disease (PD). Markerless motion capture (MMC) offers opportunities for integrating objective biomechanical outcomes into clinical practice. However, validation of MMC biomechanical outcomes is necessary for clinical adoption of MMC technologies. This project evaluated the criterion validity of a custom MMC algorithm (CART-MMC) against gold-standard 3D motion capture (Traditional-MC) and its known-groups validity in differentiating PD from healthy controls (HC). Sixty-two individuals with PD and 29 HCs completed a stepping in place paradigm. The trials were recorded by an augmented reality headset with embedded RGB and depth cameras. The CART-MMC algorithm was used to reconstruct a 3D pose model and compute biomechanical measures of lower extremity performance. CART-MMC outcomes were statistically equivalent, within 5% of Traditional-MC, for measures of step count, cadence, duration, height, height asymmetry, and normalized path length. CART-MMC captured significant between-group differences in step height, height variability, height asymmetry, duration variability, and normalized path length. In conclusion, CART-MMC provides valid biomechanical outcomes that characterize important domains of PD lower extremity function. Validated biomechanical evaluation tools present opportunities for tracking subtle changes in disease progression, informing targeted therapy, and monitoring treatment efficacy.

## 1. Introduction

Gait and postural control are early developmental milestones critical to functional mobility and independence throughout all stages of life [1]. Although automatic, effective locomotion and postural stability require complex neural and biomechanical control [2,3]. Parkinson’s disease (PD), a progressive neurodegenerative disorder, disrupts the neural circuitry involved in motor control. Postural instability and gait difficulty (PIGD) can be a profoundly debilitating PD symptom, contributing to people with Parkinson’s disease (PwPD) falling at twice the rate of healthy peers [4,5,6]. Falls increase the risks of fractures and hospitalizations, leading to increased costs of managing PD, declines in quality of life, and hastening the transition to assisted living [7,8,9]. A fundamental gap in the effective treatment of PIGD, which is often refractory to typical antiparkinsonian medication [10], is the lack of objective and scalable biomechanical analyses to inform clinical decision-making, such as tracking treatment efficacy and monitoring disease progression.

Traditional assessments of PD symptoms often rely on subjective ratings which are ill-suited for detecting subtle deficits in lower extremity function. The Movement Disorders Society-Unified Parkinson’s Disease Rating Scale, Part III (MDS-UPDRS III), the most widely used clinical scale [11,12], is susceptible to sensitivity and reliability pitfalls [13,14,15]. Despite the impact of PIGD on quality of life, only three of the thirty-three assessment items are PIGD-specific, compared to ten tremor-specific items [16]. While there are mobility-focused clinical assessments, such as the 10 m walk test and Timed Up and Go, they offer only a cursory overview of global motor performance with time to completion as the sole outcome. An isolated temporal measure of global motor performance fails to characterize subtle, nuanced mobility impairments that emerge as PD progresses [17]. Thus, objective approaches to lower extremity assessment are needed, and these approaches should be scalable if outcomes are to advance the evaluation and treatment of PIGD [18].

Three-dimensional marker-based gait and postural control analysis has long been understood as the ideal tool for comprehensive biomechanical assessment [19]. Unfortunately, the routine collection of biomechanical data has not been implemented in clinical care, even in the most advanced movement disorders centers. Traditional gold-stand motion capture (Traditional-MC) requires multiple cameras, a large open space, trained personnel, precise marker positioning, and extensive setup time [20]. Specifically, the cameras for traditional marker-based systems are sensitive to subtle movements and temperature changes and therefore must be calibrated frequently; individual markers must be hand-placed on specific anatomical landmarks; trained personnel are required to run and monitor the software, as well as manually process each individual trial. The resources required for Traditional-MC systems preclude their integration into clinical settings.

Technological advances in markerless motion capture (MMC) present an opportunity to objectively assess mobility with a low-cost portable device. The Microsoft HoloLens 2 (HL2), an augmented reality (AR) headset equipped with an RGB camera, a depth camera and spatial mapping capabilities, offers a combination of robust onboard hardware and software conducive for MMC. Camera data and images from the HL2 can be used to construct 3D position data for biomechanical analyses. Paired with MMC, a repetitive stepping in place (SIP) task can be leveraged for a detailed assessment of lower extremity function. The repetitive SIP task has been shown to elicit clinically relevant mobility impairments in PwPD, including hypokinesia, asymmetry and freezing of gait (FOG) [21,22]. Using the HL2 to quantify stepping performance offers an objective, clinically feasible paradigm for assessing PIGD symptoms.

The Comprehensive Augmented Reality Testing (CART) platform was designed for the objective assessment of PD motor symptoms, with a suite of upper and lower extremity assessment modules. The participant’s headset was used to deliver standardized instructions and project demonstrations for all assessment modules, as well as to record participant hand positions to quantify upper extremity function. For lower extremity assessments, the clinician’s HL2 captures full body movements. This manuscript focuses on the SIP module, during which the clinician records participant’s full body movement. A custom post-processing pipeline was developed to calculate a 3D pose model for biomechanical analysis. CART-MMC leverages the HL2’s onboard hardware and software and a post-processing pipeline for markerless motion capture during the CART modules. The primary aims of this project were to evaluate the criterion validity of CART-MMC outcomes from an SIP task compared to Traditional-MC and determine the known-groups validity of CART-MMC in distinguishing stepping performance between PwPD and healthy controls (HC).

## 2. Materials and Methods

### 2.1. Participants

Data sets from 62 PD and 29 age-matched HCs were analyzed. Inclusion criteria for the PD group included (1) diagnosis of idiopathic PD, (2) Hoehn and Yahr stage I–IV [23], (3) absence of a neurological condition other than PD, (4) ability to follow 2-step commands, and (5) stable antiparkinsonian medication regimen for a minimum of one month prior to assessment. Inclusion criteria for the HC group included (1) the absence of any neurological condition and (2) the ability to follow 2-step commands. For both groups, exclusion criteria included (1) implanted deep brain stimulation device, (2) history of a gait-altering musculoskeletal injury, or (3) uncorrected vision or hearing impairments that would impact interaction with an AR headset. The study was approved by the Cleveland Clinic Institutional Review Board, and all participants completed the informed consent process prior to data collection.

Data collection was completed in the off-medication state, operationally defined as at least 12 h after the participant’s last antiparkinsonian medication dose. Parkinson’s disease motor symptom severity was assessed with the MDS-UPDRS III by an MDS-certified examiner, and disease severity was classified according to the Martínez-Martín et al. criteria (mild = MDS-UPDRS III score of 0–32, moderate = 33–58, and severe = 59+) [24].

### 2.2. Stepping in Place Setup and Procedure

**Figure 1:**
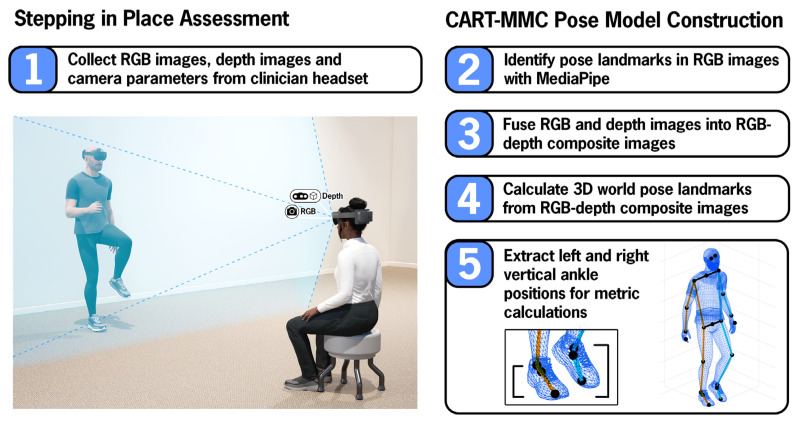
Stepping in Place Assessment: The clinician, seated approximately 10 feet away from the participant, collected RGB images, depth images, and camera parameters from their HL2. Standardized task instructions were given through the participant’s HL2. CART-MMC Pose Model Construction: Flowchart depicting the post data collection processing steps utilized for CART-MMC.

Each participant completed one 60 s SIP trial while wearing the HL2 (Microsoft, Redmond, WA, USA) headset. The participant’s HL2 delivered standardized task instructions while an AR avatar demonstrating the task was projected onto the participant’s visual display. Participants were instructed to step in place at a comfortable pace, raising their knees towards the ceiling. The clinician was seated ~10 ft away, facing the participant, wearing an HL2 used for data capture. Figure 1 depicts the data collection setup and summarizes the processing steps utilized for CART-MMC (detailed in Section 2.3).

The clinician’s HL2 captured RGB images, depth images and camera parameters used for CART-MMC. A subset of 46 participants (23 HC, 23 PD) wore an active marker set for comparison against Traditional-MC. For Traditional-MC, nineteen LED active markers (SuperNova; Vicon Motion Systems, Oxford, UK) were positioned at anatomical landmarks according to a modified Plug-in-Gait marker setup [25]. Specifically, three markers were positioned on each foot—on the lateral malleolus, the calcaneus, and the second metatarsal head and three on each leg—on the thigh, the shank, and over the lateral flexion-extension axis of the knee. Four additional markers were positioned on the hips—on the left and right anterior and posterior iliac spine, respectively. Three markers were positioned on the torso—on the clavicle, on the spinous process of the 7th cervical vertebrae, and over the right scapula. The participant wore a battery pack in a waist-worn holster that powered the markers. Traditional-MC utilized sixteen motion capture cameras (Vicon Motion Systems, Oxford, UK) to record the LED active marker positions at 100 Hz. The active markers allowed for sampling without the Traditional-MC infrared strobes enabled, preventing infrared interference with the HL2 depth camera. Data streams from the HL2 and Traditional-MC system were synchronized via trial initiation and termination triggers sent over a local network.

### 2.3. CART-MMC Human Pose Model

#### 2.3.1. CART Platform Development

The CART HL2 application was developed in Unity 2021.3.4f1 using C#, Microsoft Mixed Reality Toolkit 3.0.0-pre14, Microsoft Mixed Reality OpenXR Plugin 1.8.0, and Unity OpenXR Plugin 1.6.0. The HL2 ran the Windows Holographic operating system version 23H2, build number 22,621.1258.

#### 2.3.2. Data Collection of Images and Camera Parameters

Research Mode was used to collect HL2 camera images and camera parameters from the clinician’s headset for the CART-MMC analysis. A dynamic-link library plugin written in C++ used the Research Mode API to obtain camera related data. RGB images were collected at 30 Hz with a resolution of 1280 × 720. Depth long throw images were sampled at 5 Hz with a resolution of 320 × 288. RGB images were saved as binary data in bytes files, and depth images were saved as Portable Gray Map (PGM) files. Two tar files were created to store these RGB and depth data, respectively. In addition to the RGB and depth images, RGB and depth camera parameters were saved to text files.

The following RGB camera parameters were saved: Cam2World_RGB_, Timestamps_RGB_ and K_RGB_. Cam2World_RGB_ contained the transformation from RGB camera coordinates to world coordinates for each RGB image. Timestamps_RGB_ defined the time at which each RGB image was captured. K_RGB_ contained the intrinsics necessary for conversion of RGB camera coordinates to RGB image coordinates.

The following depth camera parameters were output from the HL2: Look-Up-Table (LUT), Rig2Cam_Depth_, Rig2World_Depth_, and Timestamps_Depth_. The Research Mode API did not provide the intrinsic parameters of the depth camera directly. Instead, the LUT contained the mapping of depth image coordinates to 3D camera unit coordinates, estimated using a Levenberg–Marquardt optimization. The Rig2Cam_Depth_ transformation was used to convert between depth camera and rig coordinates. Timestamps_Depth_ contained the timestamp for each depth image. Rig2WorldDepth contained a transformation from the rig to the world coordinate system for each depth image captured.

#### 2.3.3. Image Extraction

The CART-MMC analysis was performed offline in MATLAB (MathWorks, MATLAB R2022b). First, RGB images were extracted from the tar file into individual bytes image files, then converted to PNG images. Depth images were extracted from the tar file and converted from PGM images to PNG images.

#### 2.3.4. Identify Pose Landmark Positions in the RGB Images with MediaPipe

The RGB images were tracked using Google MediaPipe pose landmark detection software (Version 0.10.13). Thirty-three pose landmark positions, including the left and right ankle, were identified for each RGB image. The 2D positions were saved as normalized units (0, 1) and converted to the RGB image coordinate space using the RGB image width and height.

#### 2.3.5. Calculation of RGB-Depth Composite Images

For each depth image, the closest temporal RGB image was matched by the minimum absolute difference between the depth image timestamp and Timestamps_RGB_. Each set of paired images was used to calculate an RGB-depth composite image, using methods previously reported [26]. Briefly, the LUT was used to map depth image coordinates to 3D camera unit coordinates (unit vector). Equations (1) and (2) were used to convert depth camera unit coordinates into world point cloud coordinates, where ∘ represents the element-wise (Hadamard) product and d_Depth_ represents the depth value (i.e., distance from depth camera) corresponding to each depth camera unit coordinate. Variables were represented as row-wise vectors with 92,160 elements (320 × 288). Equations (3) and (4) were then used to project world point cloud coordinates into the RGB image coordinate space. World points reprojected on the RGB image that fell outside the RGB image size were considered invalid and removed. The remaining points yielded the RGB-depth composite image.

(1) $${{\left[\begin{array}{c} \vec{x}\\ \vec{y}\\ \vec{z} \end{array}\right]}_{Depth\_Cam}=\left[\begin{array}{c} \vec{x}\\ \vec{y}\\ \vec{z} \end{array}\right]}_{Depth\_CamUnit}\circ \left[\begin{array}{c} {\vec{d}}_{Depth}\\ {\vec{d}}_{Depth}\\ {\vec{d}}_{Depth} \end{array}\right]$$

(2) $${{\left[\begin{array}{c} \vec{x}\\ \vec{y}\\ \vec{z}\\ \vec{1} \end{array}\right]}_{World}=\left({Rig2World}_{Depth}\right)\times {\left({Rig2Cam}_{Depth}\right)}^{-1}\times \left[\begin{array}{c} \vec{x}\\ \vec{y}\\ \vec{z}\\ \vec{1} \end{array}\right]}_{Depth\_Cam}$$

(3) $${\left[\begin{array}{c} \vec{x}\\ \vec{y}\\ \vec{z}\\ \vec{1} \end{array}\right]}_{RGB\_Cam}={{\left({Cam2World}_{RGB}\right)}^{-1}\times \left[\begin{array}{c} \vec{x}\\ \vec{y}\\ \vec{z}\\ \vec{1} \end{array}\right]}_{World}$$

(4) $${\left[\begin{array}{c} \vec{u}\\ \vec{v}\\ \vec{z} \end{array}\right]}_{RGB\_Image}={K_{RGB}\times \left[\begin{array}{c} \vec{x}\\ \vec{y}\\ \vec{z} \end{array}\right]}_{RGB\_Cam}$$

**Figure 2:**
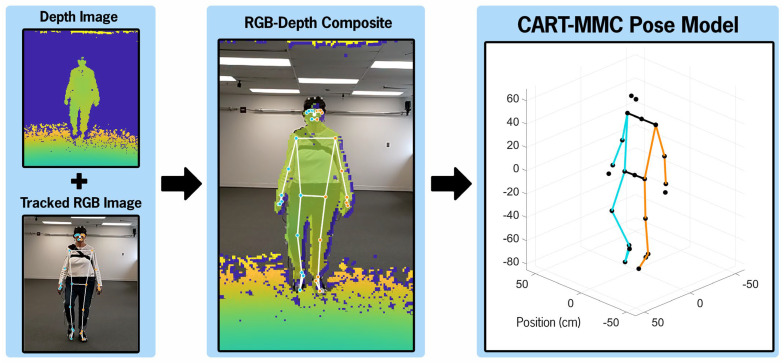
A temporally aligned depth image and Mediapipe-tracked RGB image are shown. Depth values from the depth image are projected onto the tracked RGB image, yielding an RGB-depth composite image. From the RGB-depth composite image, the CART-MMC 3D pose model is generated.

Figure 2 illustrates the process from RGB and depth images to the resultant CART-MMC 3D pose model.

The headset cameras recorded at fixed rates, 5 Hz for the depth camera and 30 Hz for the RGB camera, so corresponding depth images were present for one out of every six RGB images. For RGB images without paired depth images, depth values for each pose landmark were linearly interpolated from depth values determined from RGB-depth composite images.

#### 2.3.6. 3D World Pose Landmarks

Following depth interpolation, every RGB frame contained RGB image coordinates and corresponding depth values for each of the 33 pose landmarks. Using the MediaPipe image coordinates and corresponding depth values, camera coordinates were calculated using the inverse K_RGB_ matrix (Equation (5)), where ∘ represents the element-wise (Hadamard) product and d_RGB_ represents the depth value (distance from RGB camera). Variables are represented as row-wise vectors with 33 elements (one for each pose landmark). Three-dimensional world pose landmarks compromising the CART-MMC pose model were then computed using the Cam2World_RGB_ matrix (Equation (6)). The resultant human pose model contained 3D positions of 33 pose landmarks at 30 Hz, including the left and right ankle positions.

(5) $${\left[\begin{array}{c} \vec{x}\\ \vec{y}\\ \vec{z} \end{array}\right]}_{RGB\_Cam}={{\left(K_{RGB}\right)}^{-1}\times (\left[\begin{array}{c} \vec{u}\\ \vec{v}\\ \vec{1} \end{array}\right]}_{RGB\_Image}\circ \left[\begin{array}{c} {\vec{d}}_{RGB}\\ {\vec{d}}_{RGB}\\ {\vec{d}}_{RGB} \end{array}\right])$$

(6) $${\left[\begin{array}{c} \vec{x}\\ \vec{y}\\ \vec{z}\\ \vec{1} \end{array}\right]}_{World}={{Cam2World}_{RGB}\times \left[\begin{array}{c} \vec{x}\\ \vec{y}\\ \vec{z}\\ \vec{1} \end{array}\right]}_{RGB\_Cam}$$

### 2.4. Traditional-MC Human Pose Model

Traditional-MC data were processed with Vicon software (Nexus 2.16.0). The raw marker positions were reconstructed, and the nineteen markers were labeled for each trial. This data was then manually reviewed to ensure data integrity (e.g., complete, correctly labeled tracking) and processed with a custom Nexus pipeline (“Delete Unlabeled Trajectories”, “Process Dynamic Plug-In-Gait Model”, “Export ASCII”). The final pose model contained 3D marker positions and calculated joint center positions, including the left and right ankle joint centers.

### 2.5. Data Processing and Metric Calculations

#### 2.5.1. Signal Pre-Processing and Spatiotemporal Alignment

The same pre-processing steps were used for both the CART-MMC and Traditional-MC data. First, all CART-MMC and Traditional-MC pose landmark positions were resampled to 60 Hz using linear interpolation. The first and last 1 s of each trial were removed. Data for each pose landmark was then filtered using a 4th order Butterworth lowpass filter with a 3 Hz cutoff frequency. Because the vertical axis was common between both systems, determined through calibration for Traditional-MC and spatial mapping for CART-MMC, spatial alignment was only necessary on the anterior–posterior and medial–lateral axes (rotation around vertical axis). To spatially align the two systems, the average vector pointing from left to right hips across the entire trial was aligned to the x-axis. Specifically, the left and right hip joint centers were aligned to the x-axis for Traditional-MC and the Mediapipe defined hip positions were aligned to the x-axis for CART-MMC. Following spatial alignment, ankle position data were used to temporally align the poses by minimizing the root mean squared error between vertical ankle position data across temporal shifts. Excess data shifted to the start or end of trial due to temporal alignment were removed to guarantee both poses had the same number of samples aligned in time. A linearly interpolated envelope was fitted to minima values in vertical ankle position and subtracted off to minimize baseline noise while the ankle was planted on the ground.

#### 2.5.2. Systematic Bias Correction of CART-MMC Ankle Positions

**Figure A1:**
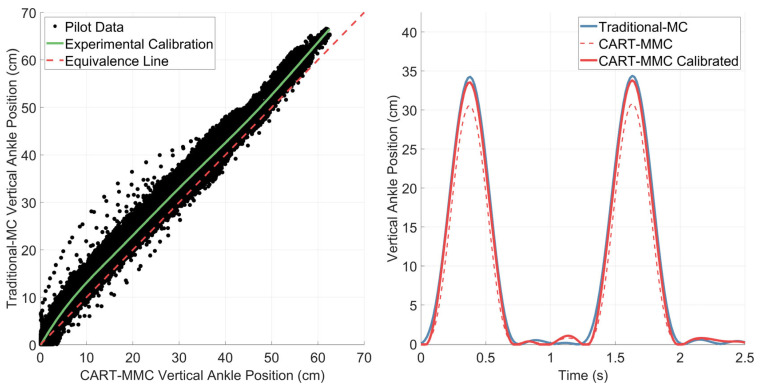
(**a**) Individual scatter points show the CART-MMC vertical ankle position versus Traditional-MC vertical ankle position for all pilot study trials. The systematic bias is characterized by a spline fit, shown in green. Vertical ankle position as measured by CART-MMC was consistently underestimated compared to Traditional-MC. (**b**) Representative ankle vertical position data for a single foot are shown both before and after systematic bias correction in comparison to Traditional-MC.

CART-MMC vertical ankle positions were corrected for systematic bias, resulting from limitations of the Mediapipe commercial pose tracking software. Based on pilot data, the MediaPipe-defined ankle positions were observed to be inconsistent with true ankle joint centers, resulting in consistent underestimation of ankle range of motion by CART-MMC (Section Pilot Data to Quantify and Correct for Systematic Bias in Pose Estimation). The CART-MMC vertical ankle position was plotted against the Traditional-MC ankle vertical position for all pilot study trials. A spline with 5 knots was fit through the data to characterize the vertical ankle position underestimation. The experimentally determined calibration curve was used to correct the CART-MMC vertical ankle position for all data presented in this manuscript (Figure A1). The following metric calculations were performed on the corrected ankle position for CART-MMC data.

#### 2.5.3. Metric Calculations

**Figure 3:**
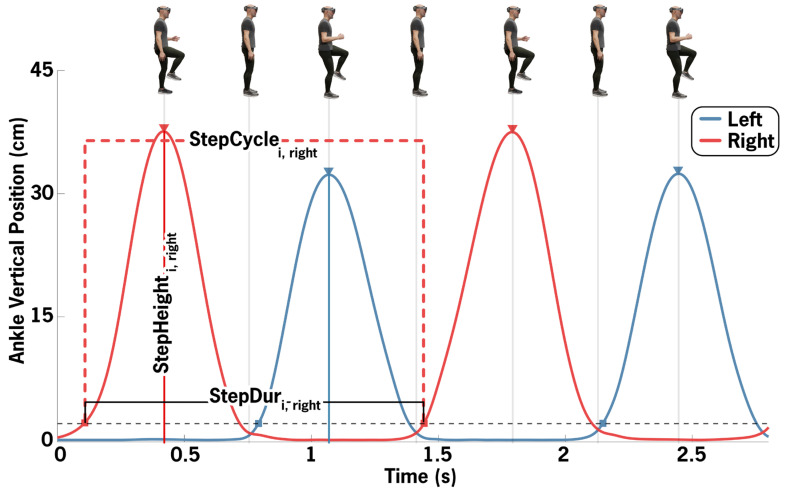
Merics from one StepCycle from the right ankle are annotated. One StepCycle includes both the swing and stance phases, from ankle off to ankle off. StepDur is the elapsed time per StepCycle. StepHeight is the maximum ankle vertical position during the swing portion.

Stepping performance was characterized by using vertical left and right ankle positions (Figure 3). Steps were defined when the ankle lifted off the ground: vertical ankle position exceeded 2.5 cm for at least 0.25 s in duration. It was assumed that only one foot was in swing phase at a single time. Therefore, if steps from both the left and right ankle were detected in the same swing phase, the lower height step was ignored. One StepCycle was defined as ankle lift off to ankle lift off. StepDur was defined as the time to complete one StepCycle. StepHeight was the maximum vertical position during the swing portion of a given cycle. If no qualifying step was detected for a duration greater than 1.5 × the median StepDur, the segment was considered a freeze. When fewer than five StepCycles were detected for a given side, that side was classified as being in a freeze state for the duration of the trial. FreezeDuration was the time within a trial spent freezing, averaged across left and right sides.

StepCount was defined as the number of step cycles completed. Cadence was defined as StepCount divided by trial duration. Mean and coefficient of variation (CV) metrics were calculated on the middle 95% of StepDur and StepHeight values combined across sides (StepDur-Mean, StepDur-CV, StepHeight-Mean, StepHeight-CV). StepDur-CV is synonymously referred to as step arrhythmicity. Normalized path length (NPL) was calculated as the sum of the absolute value of the difference between consecutive vertical ankle positions divided by trial duration. NPL was summed across left and right sides. StepHeight-Asymmetry was defined as the difference between the average larger side StepHeight and average smaller side StepHeight, divided by the average StepHeight of both sides.

### 2.6. Statistical Analysis

The goals of the statistical analysis were to (1) assess the criterion validity between CART-MMC and Traditional-MC outcomes and (2) assess the known-groups validity between PD and HC using the CART-MMC outcomes. The level of significance was held at 0.05 for statistical tests. All statistical analyses were conducted using RStudio 2025.05.0, R version 4.5.0.

#### 2.6.1. Criterion Validity Statistical Analysis

To assess criterion validity between systems, for each metric, two-one-sided t-tests (TOSTs) were conducted, using bounds of ±5% of the Traditional-MC mean value for all outcomes except for the percentage outcome, StepHeight-Asymmetry, which used ±5% overall. To additionally quantify agreement between systems, Lin’s Concordance Correlation Coefficient (Lin’s CCC) was calculated.

#### 2.6.2. Known-Groups Validity Statistical Analysis

Known-groups validity was assessed for each outcome using Welch’s *t*-tests. To further quantify the magnitude of mean differences by group, Hedges’ g was calculated.

### 2.7. Final Dataset for Analyses

Data from 91 participants (62 PD, 29 HC) were included in the analyses. Of the 101 participants who performed the SIP task, 10 were removed (6 HC, 4 PD) due to technical errors. Infrared interference occurred when the Traditional-MC infrared strobes corrupted the HL2 depth images, accounting for six of the 10 technical errors. Such an error would not be present outside of validation. Each of the four remaining errors occurred once: HL2 failed spatial mapping, HL2 file writing issue, failure of MediaPipe pose estimation and Traditional-MC tracking issue. Demographics for the final cohort are provided in Table 1.

#### 2.7.1. Criterion Validity Dataset

The equivalence analysis to evaluate criterion validity was conducted on a subset of participants (23 HC, 23 PD) who wore the active marker set, comparing the Traditional-MC outcomes to the CART-MMC outcomes.

#### 2.7.2. Known-Groups Validity Dataset

The known-groups analysis was conducted on the full set of 91 participants (29 HC, 62 PD).

## 3. Results

### 3.1. CART-MMC Outcomes Demonstrate Criterion Validity with Traditional-MC

For StepCount, Cadence, StepDur-Mean, StepHeight-Mean, NPL and StepHeight-Asymmetry, the mean difference between CART-MMC and Traditional-MC was significantly equivalent (all *p* < 0.001), within the 5% bounds (Table 2). The mean differences for StepDur-CV and StepHeight-CV were 5.7% (*p* = 0.59) and 6.7% (*p* = 0.72), respectively.

### 3.2. CART-MMC Demonstrates Known-Groups Validity

**Figure 4:**
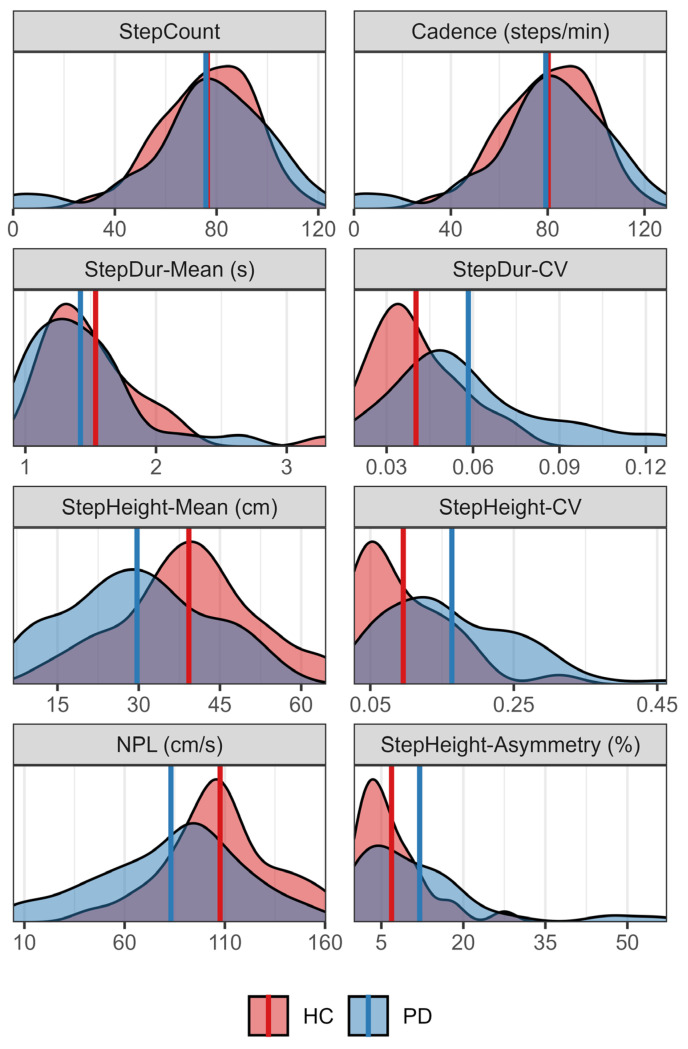
The distribution of outcomes as measured by CART-MMC for the PD (blue) and HC (red) groups. Mean values for each group are represented by vertical lines. Temporal measures of global performance, StepCount, Cadence and StepDur-Mean, are similar between groups. However, it is noteworthy that individual PD StepCount and Cadence values extend to further ends of the distributions, either a lack of stepping or faster stepping. Reduced movement is observed in the PD group compared to HCs, indicated by StepHeight-Mean and NPL. The PD group also has increased variability compared to HCs as measured by StepDur-CV and StepHeight-CV. Lastly, greater StepHeight-Asymmetry is observed for the PD group compared to HCs.

Summary statistics by group are presented in Table 3 and the distributions of each metric by group are portrayed in Figure 4. As measured by the CART-MMC, no significant differences were observed between groups for StepCount, Cadence or StepDur-Mean (all *p* > 0.24). The PD group showed a significantly greater variability in stepping compared to HCs, with StepDur-CV and StepHeight-CV 45% and 71% larger respectively (*p* < 0.001 for both). StepHeight-Mean and NPL were 24% and 23% smaller in the PD group compared to the HC group (*p* < 0.001 for both). For StepHeight-Asymmetry, the PD group showed a 74% proportional increase compared to HC, or 5.1 percentage points overall (*p* < 0.001). For the PD group, freezing occurred in 13 trials (out of 62) with a FreezeDuration mean (SD) of 22.27 s (22.16 s). Freezing was not observed in any of the 29 trials for HCs.

## 4. Discussion

The CART platform leverages a low-cost, portable AR headset to provide a valid biomechanical assessment of stepping in place. When benchmarked against a gold-standard traditional 3D motion capture system, CART-MMC demonstrated criterion validity, with outcomes deviating by 2.1% averaged across eight comprehensive biomechanical measures. Rigorous validation is an imperative step on the path to clinical integration of emerging technologies. As MMC use in clinical research expands, establishing criterion validity against gold-standard systems ensures accuracy and clinical confidence in the metrics provided [27]. Previous studies have employed closely analogous paradigms to assess stepping performance in PwPD using MMC, but without any validation against gold-standard motion capture, their accuracy and clinical relevance remain uncertain [21]. By demonstrating that CART-MMC outcomes are consistent with Traditional-MC, the present study provides the empirical foundation necessary for clinical translation.

Establishing the criterion validity of CART-MMC required correction for known limitations of pose estimation software, specifically, joint center locations that differ from true anatomical joint centers [20]. For the present study, pilot data revealed an underestimation of vertical ankle range of motion that was consistent across participants, necessitating a correction factor. An empirical correction was developed to correct for the systematic bias observed. Further, pose estimation software provides estimated depth values, shown to be unreliable [28]. Thus, using depth data from the AR headset-embedded depth camera, instead of MediaPipe native estimated depth values, provided an optimal solution.

The outcomes derived from the CART-MMC human pose model capture clinically meaningful domains of functional mobility, including pace, rhythm, asymmetry, variability and postural control [29], components of motor function known to be disrupted by PD. Compared to controls, PwPD exhibited significantly greater step duration variability, reduced step height, increased step height variability, reduced NPL, and greater asymmetry. In contrast, global measures such as step count, cadence, and step duration did not differ significantly between groups. These between-group results highlight a critical limitation of traditional observation and stopwatch-timed assessments [30,31]: global temporal metrics mask subtle but clinically relevant motor deficits, whereas CART-MMC captures the hallmark pathophysiology of PD. Specifically, reduced step amplitude and NPL reflect hypokinesia and bradykinesia, consistent with dopamine depletion [32]. Increased variability in step timing and step height reflects impaired motor regulation caused by basal ganglia dysfunction [32,33], and asymmetry reflects impaired postural control [34]. Additionally, freezing behavior was observed in 21% of participants with PD and 0% of HCs, indicating that CART-MMC may be sensitive to FOG, a profoundly debilitating PD symptom. Together, these results demonstrate known-groups validity. CART-MMC provides sensitive, clinically meaningful measures that align with the established PD motor pathology.

An evolution toward objective assessment tools such as CART-MMC and data-driven clinical decision-making, particularly for PIGD, represents a critical opportunity to improve quality of life for PwPD. Falls remain among the most disabling and life-altering consequences of PD, yet gait and postural control often respond inconsistently—and at times adversely—to dopaminergic medications and deep brain stimulation (DBS) [35,36,37,38]. Precise monitoring of PIGD is therefore essential, not only to detect treatment-related changes but also to anticipate risk before falls may occur. Traditional assessments lack the resolution to capture these subtle but clinically meaningful changes, leaving a gap that CART-MMC is uniquely positioned to fill. By identifying emerging impairments in gait patterns, CART-MMC enables refinement of therapeutic strategies and proactive referrals to physical therapy which can substantially improve patient safety via postural stability and motor control training [39]. For instance, reduced step height (i.e., foot clearance) is common in PD, is associated with increased fall risk, and is responsive to targeted rehabilitative training [40,41,42]. With the validity of CART-MMC and its lower extremity biomechanical outcomes established, future studies should evaluate its feasibility in clinical settings. Additionally, the portable, user-friendly design of the CART platform opens the possibility of home-based assessment, which can democratize high-fidelity evaluation of PD symptoms and should be investigated in future studies.

Recent advancements in AR technology, rapidly expanding commercial ecosystems, and a growing body of clinical trials demonstrating feasibility make it practical, and increasingly cost-effective, to integrate AR systems into clinical workflows [43,44,45]. The integration of MMC using an AR headset into clinical workflows offers several advantages that address longstanding barriers to comprehensive evaluation of PD [46]. The MDS-UPDRS Part III, despite its widespread use, includes only three items related to PIGD and remains subject to inter-rater variability [13,15,47]. In contrast, CART-MMC delivers objective, high-resolution data capable of detecting subtle impairments that traditional scales often miss. Importantly, PIGD symptoms are often refractory to traditional PD therapies [33,48]. Because tremor-related improvements can mask persistent gait deficits, or even declines, reliance on the MDS-UPDRS III alone may obscure clinically meaningful change. By supplementing traditional evaluations, CART-MMC can provide a comprehensive, objective approach to assessing PD motor symptoms—informing targeted therapy, monitoring treatment efficacy, and tracking disease progression with greater sensitivity [49].

## 5. Conclusions

In summary, CART-MMC offers a valid, portable solution for high-resolution biomechanical assessment in PwPD. Its ability to deliver accurate, objective, clinically relevant data marks a significant advance in mobility evaluation, and its low-cost, portable hardware makes it feasible for clinical integration. The adoption of rigorously validated platforms like CART-MMC can provide data to help clinicians in performing accurate, objective assessments, guiding personalized interventions, and ultimately enhancing outcomes for individuals living with Parkinson’s disease.

## Figures and Tables

**Table 1:** Participant demographics.

	Healthy Controls (*n* = 29)	Parkinson’s Disease (*n* = 62)
**Age (yrs)**	66.4 (6.94)	67.9 (7.81)
**Gender**		
Female	16 (55.2%)	14 (22.6%)
Male	13 (44.8%)	48 (77.4%)
**Race**		
White	29 (100%)	58 (93.5%)
Black	0 (0%)	4 (6.5%)
**Education (yrs)**	17.8 (3.95)	17.0 (2.21)
**MDS-UPDRS III Motor Score**	-	36.6 (13.2)
**Severity Score**		
Mild	-	24 (38.7%)
Moderate	-	34 (54.8%)
Severe	-	4 (6.5%)

Data are summarized as mean (SD) or *n* (%).

**Table 2:** CART-MMC outcomes demonstrate equivalence to Traditional-MC.

Outcome	Traditional-MC Mean (SD)	CART-MMC Mean (SD)	% Difference	Equivalence *p*-Value	Lin’s CCC
StepCount	74.52 (24.51)	74.30 (24.47)	−0.3%	<0.001 **	0.998
Cadence (steps/min)	78.20 (25.54)	77.97 (25.51)	−0.3%	<0.001 **	0.998
StepDur-Mean (s)	1.51 (0.44)	1.51 (0.44)	0.0%	<0.001 **	1.000
StepDur-CV	0.046 (0.022)	0.049 (0.025)	5.7%	0.594	0.933
StepHeight-Mean (cm)	34.70 (12.24)	34.68 (12.71)	0.0%	<0.001 **	0.990
StepHeight-CV	0.114 (0.071)	0.122 (0.078)	6.7%	0.716	0.948
NPL (cm/s)	90.77 (33.81)	92.62 (34.80)	2.0%	<0.001 **	0.986
StepHeight-Asymmetry (%)	10.17 (8.53)	8.35 (7.59)	−1.8% ^A^	<0.001 **	0.801

^A^ Evaluated as the difference in percentages instead of proportional percentage change, ** *p* < 0.001.

**Table 3:** CART-MMC detects stepping impairments in PwPD.

Outcome	HC Mean (SD)	PD Mean (SD)	Hedges’ g	*p*-Value
StepCount	76.38 (17.40)	75.81 (25.86)	−0.03	0.901
Cadence (steps/min)	80.21 (18.08)	79.22 (27.00)	−0.04	0.837
StepDur-Mean (s)	1.54 (0.45)	1.42 (0.39)	−0.27	0.244
StepDur-CV	0.040 (0.015)	0.058 (0.026)	0.84	<0.001 **
StepHeight-Mean (cm)	39.26 (12.46)	29.69 (12.88)	−0.75	0.001 *
StepHeight-CV	0.096 (0.067)	0.164 (0.092)	0.83	<0.001 **
NPL (cm/s)	107.63 (27.20)	83.10 (35.58)	−0.77	<0.001 **
StepHeight-Asymmetry (%)	6.87 (6.19)	12.00 (12.74)	0.51	0.013 *

* *p* < 0.05, ** *p* < 0.001.

## Data Availability

The data presented in this study are available on request from the corresponding author.

## Abbreviations

The following abbreviations are used in this manuscript:

PD	Parkinson’s disease
MMC	Markerless motion capture
HC	Healthy control
RGB	Red, green, blue
PIGD	Postural instability and gait difficulty
PwPD	People with Parkinson’s disease
MDS-UPDRS III	Movement Disorders Society-Unified Parkinson’s Disease Rating Scale, Part III
HL2	HoloLens 2
AR	Augmented reality
SIP	Stepping in place
FOG	Freezing of gait
CART	Comprehensive Augmented Reality Testing
API	Application programming interface
PGM	Portable gray map
LUT	Look-Up-Table
CV	Coefficient of variation
NPL	Normalized path length
CCC	Concordance correlation coefficient
SD	Standard deviation
DBS	Deep brain stimulation

## Appendix A

### Pilot Data to Quantify and Correct for Systematic Bias in Pose Estimation

Briefly, eight PD and eight HC completed the SIP protocol with the HL2 and the Traditional-MC as described. The CART-MMC vertical ankle position was plotted against the Traditional-MC ankle vertical position for all pilot trials (Figure A1a). A clear underestimation of vertical position was observed for CART-MMC as compared to Traditional-MC, characterized by a spline fit with five knots. The experimentally determined calibration curve was used to correct CART-MMC vertical ankle position (Figure A1b).
